# 3D Complex: A Structural Classification of Protein Complexes

**DOI:** 10.1371/journal.pcbi.0020155

**Published:** 2006-11-17

**Authors:** Emmanuel D Levy, Jose B Pereira-Leal, Cyrus Chothia, Sarah A Teichmann

**Affiliations:** Medical Research Council Laboratory of Molecular Biology, Cambridge, United Kingdom; Columbia University, United States of America

## Abstract

Most of the proteins in a cell assemble into complexes to carry out their function. It is therefore crucial to understand the physicochemical properties as well as the evolution of interactions between proteins. The Protein Data Bank represents an important source of information for such studies, because more than half of the structures are homo- or heteromeric protein complexes. Here we propose the first hierarchical classification of whole protein complexes of known 3-D structure, based on representing their fundamental structural features as a graph. This classification provides the first overview of all the complexes in the Protein Data Bank and allows nonredundant sets to be derived at different levels of detail. This reveals that between one-half and two-thirds of known structures are multimeric, depending on the level of redundancy accepted. We also analyse the structures in terms of the topological arrangement of their subunits and find that they form a small number of arrangements compared with all theoretically possible ones. This is because most complexes contain four subunits or less, and the large majority are homomeric. In addition, there is a strong tendency for symmetry in complexes, even for heteromeric complexes. Finally, through comparison of Biological Units in the Protein Data Bank with the Protein Quaternary Structure database, we identified many possible errors in quaternary structure assignments. Our classification, available as a database and Web server at http://www.3Dcomplex.org, will be a starting point for future work aimed at understanding the structure and evolution of protein complexes.

## Synopsis

The millions of genes sequenced over the past decade correspond to a much smaller set of protein structural domains, or folds—probably only a few thousand. Since structural data is being accumulated at a fast pace, classifications of domains such as SCOP help significantly in understanding the sequence–structure relationship. More recently, classifications of interacting domain pairs address the relationship between sequence divergence and domain–domain interaction. One level of description that has yet to be investigated is the protein complex level, which is the physiologically relevant state for most proteins within the cell. Here, Levy and colleagues propose a classification scheme for protein complexes, which will allow a better understanding of their structural properties and evolution.

## Introduction

Most proteins interact with other proteins and form protein complexes to carry out their function [1]. A recent survey of ∼2,000 yeast proteins found that more than 80% of the proteins interact with at least one partner [2]. This reflects the importance of protein interactions within a cell. It is therefore crucial to understand the physicochemical properties as well as the evolution of interactions between proteins.

The Protein Data Bank (PDB) [3] makes available a large number of structures that effectively provide a molecular snapshot of proteins and their interactions, at a much greater level of detail than other experimental methods. In this study, we focus on X-ray crystallographic structures that represent the vast majority of all structures. Since half of the crystallographic structures are homo- or heteromeric protein complexes, crystallographic data represent an important source of information to study the molecular bases of protein–protein interactions, and more generally of protein complex formation.

To facilitate understanding of, and access to, the constantly growing body of information available on protein structures, a hierarchical classification of protein complexes is needed in the same way that SCOP [4] and CATH [5] provide a classification of protein domains. We approach this by organising complexes first in terms of topological classes, in which each polypeptide chain is represented as a point, and only the pattern of interfaces between chains is considered. Then we subdivide these classes by considering the structures and later the sequences of the individual subunits.

To our knowledge, all previous classifications have considered parts of structures rather than whole complexes. For instance, in SCOP [4] and CATH [5], proteins are divided into their structural (CATH) and evolutionary (SCOP) domains, which are subsequently classified according to their structural homology with other domains. Because domains interact with each other, both within and between polypeptide chains, domain–domain interfaces are classified in databases such as SCOPPI [6], 3did [7], iPfam [8], PSIBASE [9], and PIBASE [10].

Protein complexes, however, often contain more than two domains: they may contain multiple polypeptide chains, and each chain can contain more than one domain. Therefore, properties that depend on the whole protein complex cannot be studied by consideration of interacting domain pairs alone. Such properties of protein complexes are size, symmetry, evolution, and assembly pathway. There have been studies on manually curated subsets that address issues such as evolution of oligomers [11,12], biochemical and geometric properties of protein complexes [13], or the assembly pathways in multisubunit proteins [14,15]. The largest set of complexes in any of these references appears to be about 455 in Brinda and Vishveshwara, so none of these studies has focused on *all* known structures from a *whole protein complex* perspective.

For historical, medical, or other scientific reasons, the PDB is highly redundant, and some structures such as the phage T4 lysozyme are present in hundreds of copies. To our knowledge, no method allows the removal of redundancy among protein complexes. Available methods would break them down into nonredundant sets of domains (ASTRAL) [16], polypeptide sequences (ASTRAL), or domain pairs (SCOPPI, 3did). Therefore, none of these methods allows us to answer a question as simple as, “How many different protein complexes are there in the PDB?”

**Figure 1:**
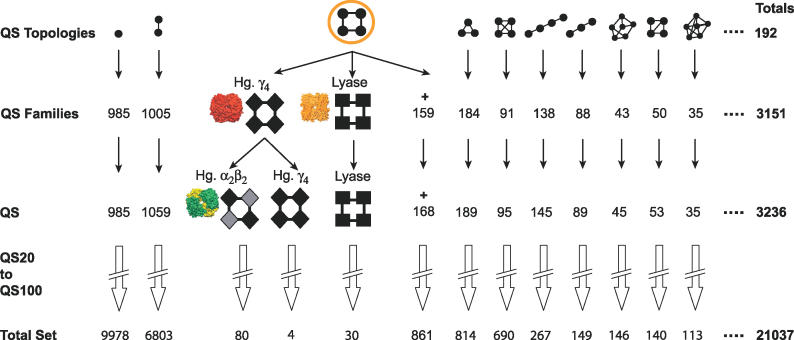
A Hierarchy of Protein Complexes of Known Three-Dimensional Structure The hierarchy has 12 levels, namely, from top to bottom: QS topology, QS family, QS, QS20, QS30…QS100. At the top of the hierarchy, there are 192 QS topologies. One particular QS topology (orange circle) with four subunits is expanded below. It comprises 161 *QS families* in total, of which two are detailed: the E. coli lyase and the H. sapiens hemoglobin γ_4_. All complexes in the E. coli lyase QS family are encoded by a single gene and therefore correspond to a single QS. However, the hemoglobin QS Family contains two QSs: one with a single gene, the hemoglobin γ_4_, and one with two genes, the hemoglobin α_2_β_2_ from H. sapiens. The last level in the hierarchy indicates the number of structures found in the complete set (PDB). There are 30 redundant complexes corresponding to the lyase QS, four corresponding to the hemoglobin γ_4_ QS, and 80 to the hemoglobin α_2_β_2_ QS. We also see that there are 9,978 monomers, 6,803 dimers, 814 triangular trimers, etc. Note that there are intermediate levels using sequence identity thresholds (fourth to twelfth level) between the QS level and the complete set, which are not shown in detail here.

**Figure 2:**
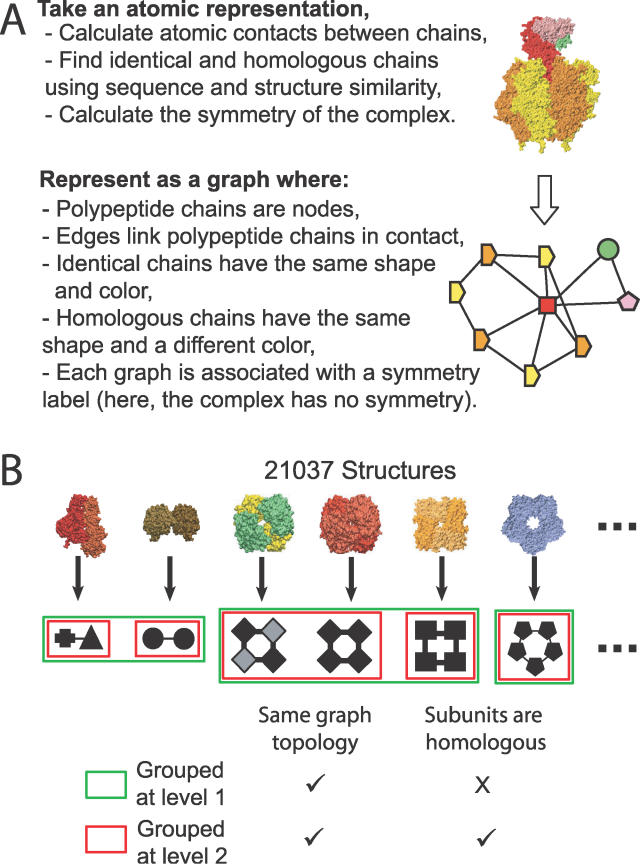
Representing Protein Complexes as Graphs (A) Each protein complex is transformed into a graph where nodes represent polypeptide chains and edges represent biological interfaces between the chains. (B) All complexes are compared with each other using a customized graph-matching procedure. Complexes with the same graph topology are grouped to form the top level of the hierarchy, as shown by the green boxes. If, in addition, the subunit structures are related by their SCOP domain architectures, they are grouped at the second level, shown by the red boxes. Structures were rendered with VMD [51].

Our structural classification of whole protein complexes (Figure 1) includes a novel strategy of visualization and comparison of complexes (Figure 2). We use a simplified graph representation of each complex, in which each polypeptide chain is a node in the graph, and chains with an interface are connected by edges. We compare complexes with a customized graph-matching procedure that takes into account the topology of the graph, which represents the pattern of chain–chain interfaces, as well as the structure and sequence similarity between the constituent chains. We use these properties to generate a hierarchical classification of protein complexes. It provides a nonredundant set of protein complexes that can be used to derive statistics in an unbiased manner. We illustrate this by drawing on different levels of the classification to address questions related to the topology, the symmetry, and the evolution of protein complexes.

## Results/Discussion

### A Dataset of Protein Complexes

We retrieved all Biological Units from the PDB (October 2005), which are the protein complexes in their physiological state, according to the PDB curators. This information is attained by a combination of statements from the authors of the structures, literature curation, and the automatic predictions made by the Protein Quaternary Structure (PQS) server [17,18]. The PDB Biological Unit is explained in more detail in Protocol S1. Inferring the Biological Unit from a crystallographic structure is a difficult, error-prone process [17,19,20]. In Ponstingl et al. (2003), an automatic prediction method was estimated to have a 16% error rate. We discuss later how our classification of protein complexes can facilitate this process and how we used it to pinpoint possible errors in Biological Units.

We filtered Biological Units according to the following criteria: we only considered the structures present in SCOP 1.69 [4] because our methodology requires SCOP superfamily domain assignments. We removed virus capsids and any complex containing more than 62 protein chains because PDB files cannot handle more than 62 distinct chains references (a–z, A–Z, 0–9), and also because of the high computational cost. We discarded structures that were split into two or more complexes when removing nonbiological interfaces as defined in the next section. When two or more copies of a complex are present in the asymmetric unit, the PDB curators create many copies of the same Biological Unit. In these cases, we retain only one copy.

After applying these filters, we obtained 21,037 structures, which we use throughout this study.

### Extracting Fundamental Structural Features from Protein Complexes

A prerequisite for creating a hierarchical classification of protein complexes is a fast way of comparing complexes with each other. The full atomic representation is not practical, because automatic structural superposition is difficult, if not impossible, for divergent pairs of structures [21]. Instead, we need to summarize the fundamental structural features of protein complexes into a representation easier to manipulate.

Which subset of features shall we choose? A natural way to break down a complex is into its constituent chains, each of which is a gene product. The pattern of interactions between the chains determines the QS and hence function of the complex. Unlike large-scale proteomic experiments, where complexes consist of a list of constituent subunits, PDB structures provide us with the QS: the exact stoichiometry of the subunits and the pattern of interfaces between them. The QS often plays a role in regulating protein function, and its disruption can be associated with diseases [22,23]. For example, in the case of the superoxide dismutase, the disruption of the QS destabilizes the protein and is linked with a neuropathology [23].

To extract the pattern of interfaces from the structures, we calculate the contacts between pairs of atomic groups. We define a protein–protein interface by a threshold of at least ten residues in contact, where the number of residues is the sum of the residues contributed to the interface by both chains. A residue–residue contact is counted if any pair of atomic groups is closer than the sum of their van der Waals radii plus 0.5 Å [24]. We investigated the effect of changing the threshold of ten residues at the interface and found that it had only a minor effect on the classification. Please refer to Table S1 for details.

As one of our goals is to compare the evolutionary conservation of protein chains both within and across complexes, we must include information that allows us to relate the chains to each other. To do this, we use structural information, as defined by the SCOP superfamily domains, as well as sequence information. The N- to C-terminal order of SCOP superfamily domains enables us to detect distant relationships, while the sequence similarity allows comparisons at a finer level, e.g., filtering of identical chains.

We chose the chain domain architecture, the sequence, and the chain–chain contacts to represent protein complexes because these are universal attributes of complexes. In contrast, other attributes such as the presence of a catalytic site, or the transient or obligate nature of an interface, are neither universal nor always available from the structure. However, these attributes can be easily projected onto our classification scheme to see how they relate among protein complexes sharing evolutionarily related chains.

To this core representation we add symmetry information, which refines the description of the subunits' arrangement beyond the interaction pattern. We process the symmetry of each complex using an exhaustive search approach. Briefly, we centre the coordinates of the complex on its centre of mass; we then generate 600 evenly spaced axes passing through the centre of mass. We check whether the complex, rotated at different angles around each of the axes, superposes onto the unrotated complex. From this, we deduce the symmetry type. For a more detailed description, please refer to the Methods section and to Figure S1.

A graph is simple and well-suited to store and visualize this information (Figure 2A). The graph itself provides what we call the topology of the complex, i.e., the number of polypeptide chains (nodes) and their pattern of interfaces (edges). A label on the graph carries the symmetry information. A label on each edge indicates the number of residues at the interface. Two further pieces of information are associated with each node in the graph: the amino acid sequence and the SCOP domain architecture of the chain. These two attributes provide information on the sequence and structural similarity and evolutionary relationships between chains. We then compare graph representations of complexes to build the hierarchical classification.

Note that we also include monomeric proteins in the classification, and we represent them by a single node. Though monomeric proteins are not complexes, their inclusion allows us to compare their frequency and other properties to those of protein complexes.

### Comparison of Complexes and Overview of the Classification

An advantage of the graph representation is that it allows fast and easy comparison using a graph-matching algorithm. As the graphs carry specific attributes about the structure and sequence of the chains, and about the symmetry of the complex, we had to implement a customized version of a graph-matching procedure to take this information into account. For algorithmic details please refer to the Methods section.

Importantly, our graph-matching procedure allows different attributes to be considered, as illustrated in Table 1 with “Y” and “N” tags. The table shows that the 12 levels of the hierarchical classification are created using one or more of the following five criteria to compare the complexes with each other: (i) the topology, represented by the number of nodes and their pattern of contacts, (ii) the structure of each constituent chain in the form of a SCOP domain architecture, (iii) the number of nonidentical chains per domain architecture within each complex, (iv) the amino acid sequence of each constituent chain for comparison between complexes, and (v) the symmetry of the complex.

**Table 1:**
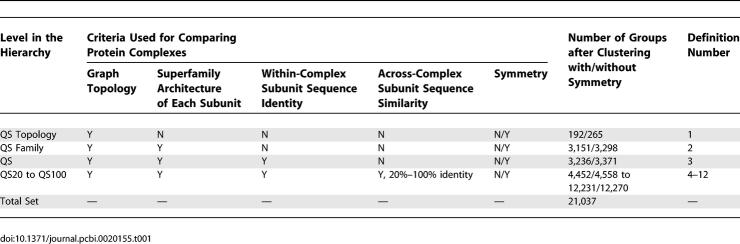
Criteria for Comparison and Classification of Protein Complexes

With these five criteria, we elaborate progressively stricter definitions of similarity between complexes as shown in Table 1. The first definition, which is the most lenient, is based solely on the topology of the graphs. This means that any two complexes with the same number of chains (nodes) and the same pattern of contacts (edges) belong to the same group, even if their chains are structurally unrelated. We use this definition to create the groups that form the top level of the classification, and we call these groups Quaternary Structure Topologies (QS Topologies, or QSTs), and we find 192 of them in the current dataset.

From the second level of the classification downward, we include evolutionary relationship information. The definition of evolutionary relationship that we use at this level is that pairs of matching nodes (polypeptides) between two graphs must have similar 3-D structures, i.e., the same SCOP domain architecture. This means that two matching polypeptide chains sometimes have little or no sequence identity, but have only structural similarity, i.e., they are distantly homologous. The groups of complexes at this level of the classification are called QS families, and we find 3,151 of them in the PDB.

At the next level, we require in addition that two matching complexes must have the same number of genes coding for each domain architecture. This is illustrated in Figure 1 where the hemoglobin QS Family splits into two groups: one containing the hemoglobin γ_4_ formed by a single gene, and one containing the hemoglobin α_2_β_2_ formed by two homologous genes. We call the groups at this level QSs. Throughout the study, we use this level composed of 3,236 QSs *as a reference set of nonredundant protein complexes*. Note that our choice for using this level as a nonredundant set is related to our interest in gene duplication events, but other levels can be used, depending on the question asked.

From level four downward, we group protein complexes according to the sequence similarity between the matching polypeptides of two complexes, from 20% identity at the fourth level to 100% at the twelfth level. We call these groups QS20/30/40, etc. As the sequence similarity threshold gets stricter, the 3,236 QS groups break down into smaller subgroups, from 4,452 QS20 at the fourth level to 12,231 QS100 at the twelfth level.

In addition to the four criteria described above, we can impose the requirement that two complexes must have the same symmetry type to be part of the same group. Because this choice is made for all levels, two classifications are available: one where symmetry is used during the comparison process and one where it is not used. When symmetry is used, we split any group of protein complexes into two or more groups, so that all complexes within a group have the same symmetry. However, only a few groups had to be split according to symmetry, as we show later.

The hierarchical classification is illustrated in Figure 1. The first three levels correspond to definitions 1 to 3 in Table 1. Note that in Figure 1, “QS20 to QS100” represents sublevels of QSs that are not illustrated but will be discussed below. The last level corresponds to the complete PDB dataset. In the next three sections, we describe the first three levels in more detail and illustrate their utility to address a variety of questions about protein complexes.

### Quaternary Structure Topologies (Level 1)

**Figure 3:**
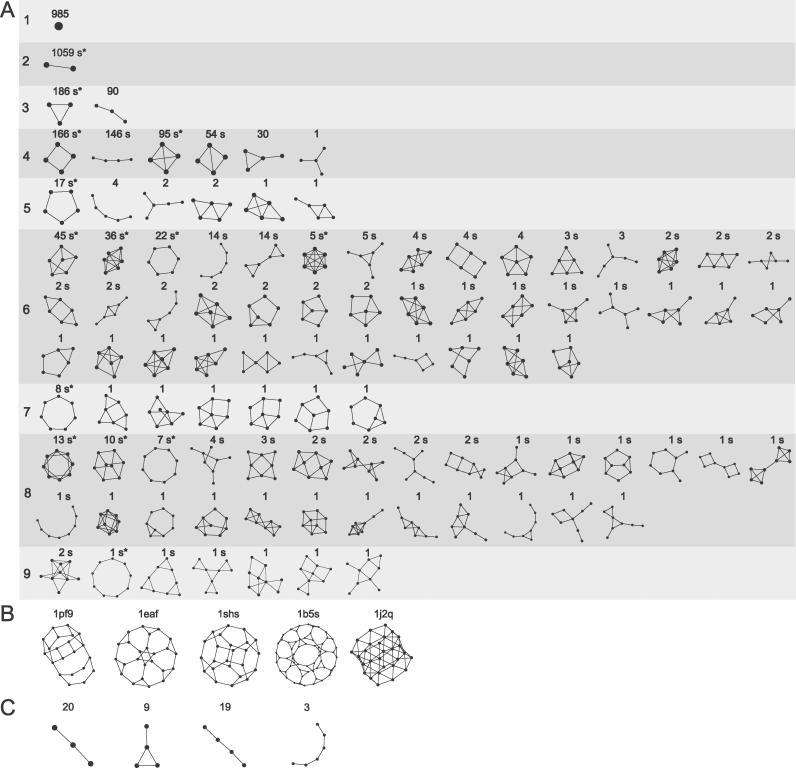
Examples of Quaternary Structure Topologies (A) All QSTs for complexes with up to nine subunits are shown, accounting for more than 96% of the nonredundant set of QSs and more than 98% of all complexes in PDB. Topologies compatible with a symmetrical complex are annotated with an *s,* and topologies where all subunits have the same number of interfaces (edges) are annotated by a star (*). (B) Examples of large complexes that are the single representatives of their respective topologies (QSTs). PDB codes are given. 1pf9, *E. coli* GroEL-GroES-ADP; 1eaf, synthetic construct, pyruvate dehydrogenase; 1shs, Methanococcus jannaschii small heat shock protein; 1b5s, Bacillus stearothermophilus dihydrolipoyl transacetylase; 1j2q, Archaeoglobus fulgidus 20S protesome alpha ring. It is interesting to note that the graph layouts resemble the spatial arrangements of the subunits. (C) Likely errors in the PDB Biological Units: QSTs of homomers with different numbers of contacts amongst the subunits. The number of erroneous QSs in each topology is provided above each graph.

QS topologies represent the number of subunits (nodes) in a complex and the pattern of interfaces (edges) between them, and is thus a topological level only. In mathematical terms, a QS topology is an unlabelled connected graph. The number of possible graphs for a given number of nodes *N* can be calculated and increases dramatically with *N* [25]*.* A single QS topology exists for *N* = 1 or *N* = 2, while there are 6 QS topologies for *N* = 4, and 261,080 for *N* = 9. Comparatively, we observe a low number, 192 QS topologies in total, that account for the 21,037 protein complexes. This low number suggests that some QS topologies are preferred over others in the protein universe. All the QS topologies containing up to nine chains are shown in Figure 3, and the number above each QST indicates the number of QSs (nonredundant structures) it corresponds to. A visual inspection of the QSTs suggests three main constraints limiting their number.

**Figure 4:**
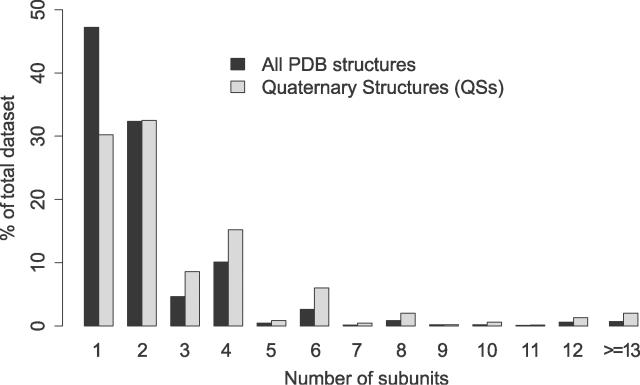
Distribution of Protein Complex Size in the Hierarchy Histogram of the number of subunits per protein complex. Smaller complexes are more abundant than larger complexes, and complexes with even numbers of subunits tend to be more abundant than complexes with odd numbers of subunits, at both levels of the hierarchy.

The first, as shown in Figure 4, is that most complexes contain a small number of chains and can therefore adopt a very restricted number of topologies. In the PDB set, we observe a sharp decrease in the proportion of complexes as the number of their chains increases, so that 94% of the structures contain four chains or less and are found in ten QSTs only.

The second constraint limiting the number of QS topologies lies in the composition of the PDB, which consists mainly of homo-oligomeric complexes, that is, complexes formed by multiple copies of the same protein. As protein–protein interfaces are often hydrophobic [26,27], a different number of interfaces in two identical proteins implies that a hydrophobic surface is exposed to the solvent in one of them, which would be unfavourable for the stability of the protein. So, in homomeric complexes, we expect all the chains to have the same number of interfaces. Excluding monomers, we observe that this is the case for 96% of homomeric complexes that represent 41% of the entire PDB. In the 4% of the cases where this criterion is not met, we observe a large proportion of erroneous QSs, as discussed below. Purely homomeric complexes are then very restricted in their topology, because for a complex with *N* chains (*N* ≥ 3), there are only *N-*2 topologies with the same number of interfaces per chain*.* For instance, only seven topologies satisfy this criterion for *N* = 9, a small number compared with 261,080 possible ones. Figure 3A shows that the most populated topologies are the ones for which all subunits have the same contact pattern, and these are marked with a star.

The third constraint limiting the number of QSTs is that 85% of the complexes in the PDB are symmetrical. This trend is captured in Figure 4, showing that complexes with even numbers of subunits are favoured, but is more explicit when looking at the QSTs in Figure 3. The graph representation reflects the possibility of presence or absence of symmetry. For all numbers of subunits, the QS topologies that are compatible with a symmetrical complex (marked with “s”) are those that are most commonly found. For example, six QS topologies are found in tetramers, and the four most common are compatible with symmetry, while the two less common are not.

So the QS Topologies allow a survey of the organization of the chains in protein complexes. This is best illustrated by the large protein complexes shown in Figure 3B, where the graph representation hints at the 3-D structure. This representation highlights that protein complexes in PDB tend to satisfy three criteria: they are predominantly small, homomeric, and symmetrical, which drastically limits the QS Topologies compared with all possible graph topologies. This result carries potential predictive power and could be used as constraints for the prediction of the topology of large assemblies [28]. Also, we will assess below to what extent this result, observed on the subset of proteins present in PDB, can be generalized to SwissProt proteins [29].

### Quaternary Structure Families (Level 2)

When we consider structural similarity in the form of domain architecture identity between pairs of matching subunits of two complexes, the 192 QSTs break down into 3,151 Quaternary Structure Families (QSFs) (Table 1, definition 2). For example, in Figure 1, an orange circle highlights a tetrameric QST that breaks down into 161 QSFs*,* two of which are shown: Escherichia coli lyase and Homo sapiens hemoglobin.

In the next level of the classification, the QS level (level 3), a constraint will be added on the number of genes per domain architecture (Table 1, definition 3). For example, the H. sapiens hemoglobin QSF will break down into two QSs: (i) the γ_4_ hemoglobins (formed by four copies of a single gene), and (ii) the α_2_β_2_ hemoglobins (formed by two copies of two homologous genes). However, all structures present in the E. coli lyase QSF consist of one gene only, and, therefore, the QSF contains a single QS.

The QSF level can be used to address questions related to the evolution of protein complexes, in particular the role of gene duplication. Each QSF that corresponds to two or more QSs points to complexes with a similar structure but a different number of genes, i.e., complexes that underwent an internal gene duplication [30–33]. This type of event is rare in PDB: 83 QSFs correspond to two QSs, as for the hemoglobins, and one QSF corresponds to three QSs, while the other 3,070 QSFs correspond to a single QS.

### Quaternary Structures (Level 3)

We have seen above that there are few QSFs that correspond to multiple QSs, so that the number of QSs is similar to that of QSFs. There are 3,236 QSs in the PDB. Some of them correspond to multiple redundant structures in the PDB. For example, Figure 1 shows that 30 structures correspond to the E. coli lyase QS, four correspond to the hemoglobin γ_4_ QS, and 80 correspond to the hemoglobin α_2_β_2_ QS. In Table 2, we list 12 QSs containing the largest number of redundant protein complexes in the PDB. Immunoglobulins and HIV-1 proteases are the most redundant with 281 and 202 complexes, respectively, in the complete PDB. The QS level represents a nonredundant version of the PDB, where cases like those illustrated in Table 2 are reduced to a single entry.

**Table 2:**
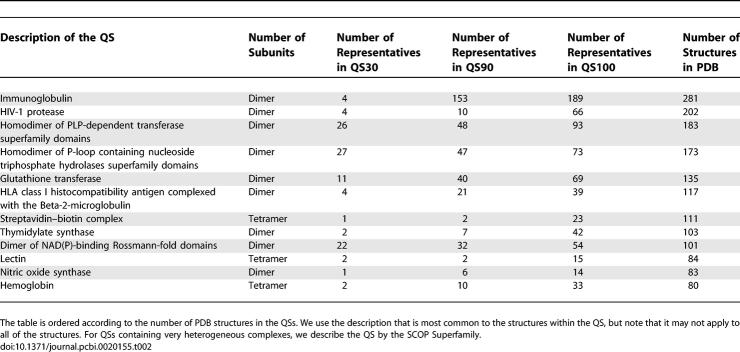
Twelve Largest Quaternary Structures with Two or More Subunits

In the structural classification of proteins SCOP, proteins with the same superfamily domains are thought to originate from a common ancestor and thus to be evolutionary related. Similarly, in the 3D Complex classification, protein complexes grouped in the same QS share evolutionarily related proteins. However, it is not known whether the entire complexes are evolutionarily related, i.e., whether their ancestral proteins interacted in the same manner. So it is important to note that within the same QS, proteins of two different complexes, even though evolutionarily related, could in principle interact in different ways, i.e., with interfaces on different surfaces of the structure. One example is the different dimerization modes of lectins discussed in [34]. However, if one does want to minimize differences in interface geometry, we provide two ways of achieving this: constraining by sequence similarity or by symmetry. For complexes with sequence identity above 30% to 40%, recent work suggests that differences in interface geometry will be rare [35]. The levels below the QS level use sequence similarity for comparing complexes and are discussed in the next section.

Note also that grouping proteins with different interaction modes does not affect the use of QSs as a nonredundant representation of the PDB. In contrast, the groups formed at the QS level can be used for studying the conservation of interactions in protein complexes, with respect to their size, place, shape, or chemical nature. In this paper, we illustrate the use of the QSs as a nonredundant set to survey the distribution of protein complex size as well as the relative abundances of their topologies as described above. We will also use it later to compare the size distribution of homo-oligomers in the PDB and in the SwissProt database. Many other studies could be carried out; for example, this level could be used to examine the diversity of oligomeric states per domain family or domain architecture.

### Adding Sequence Similarity Information to the Classification (Levels 4 to 12)

To add constraints on sequence similarity, we require a sequence identity threshold for matching pairs of proteins in our graph-matching criteria from level 4 to level 12 as indicated in Table 1. We start with a 20% identity threshold, yielding 4,452 groups, and increase it in 10% increments, to reach 12,231 groups at a threshold of 100% identity. We call the groups QS*N,* where *N* denotes the percentage identity threshold used.

**Figure 5:**
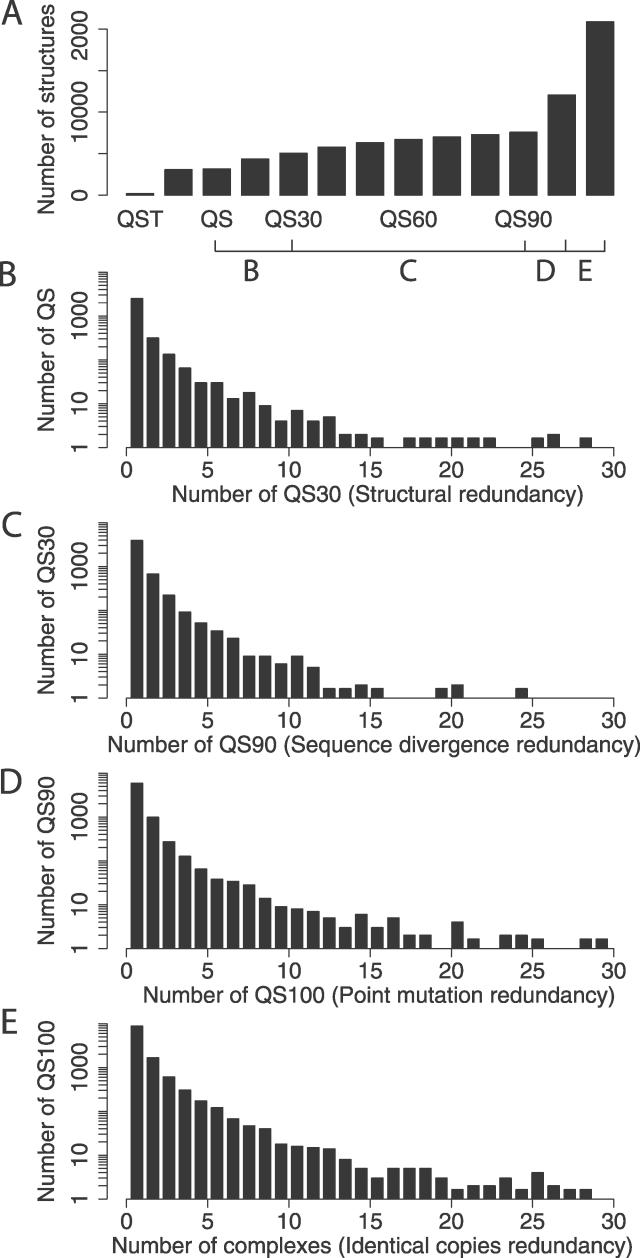
Redundancy in the Protein Data Bank at Several Levels of Sequence Similarity (A) The number of structures at each level of the 3D Complex database, from 192 QSTs to the total number of structures in the PDB (21,037). The tick marks on the line below the graph indicate the consecutive pairs of levels that are plotted in (B–E). (B) Number of QS30 per QS. Note that QS Families are almost identical to QSs. The first bar in the histogram shows that about 2,500 QS correspond to one QS30; the second bar represents 250 QS that correspond to two QS30. (C) Number of QS90 per QS30. (D) Number of QS100 per QS90. (E) Number of complexes in the complete set per QS100. All distributions display scale-free behaviour, in the sense that a large proportion of groups are identical at any two consecutive levels, whereas a small number are very redundant. Adding symmetry information does not change this trend, as shown in Table 1.

The numbers of groups for the different levels of the classification are shown in Figure 5A. The increase from the 3,236 QSs to the 21,037 complexes in the total set is not linear; instead it can be decomposed into four phases: (i) a burst in the number of groups between QSs (3,236) and QS30 (5,136), (ii) a progressive increase between QS30 and QS90 (7,713), (iii) a sharp increase between QS90 and QS100 (12,231), and (iv) a dramatic burst between QS100 and the entire PDB set (21,037).

Figure 5B–5E shows the distribution of the redundancy among these four pairs of levels. For example, Figure 5B indicates that ∼2500 QSs correspond to a single QS30, ∼300 QSs split into two QS30, ∼150 QSs split into three QS30, two QSs split into fifteen QS30, etc. This shows that most protein complexes grouped together in the same QS, on the basis of structural similarity, show sequence similarity levels above 30% for all of their chains, while a few show lower sequence similarity levels. Strikingly, the distribution of the redundancy between subsequent pairs of QS levels, such as QS30 to QS90 and QS90 to QS100, mirrors that of the QSs to QS30 even though the origins of the redundancy are unrelated. For example, the distribution between the QS30 and QS90 reflects moderate sequence divergence between related complexes. The redundancy observed between the QS90 and QS100 essentially corresponds to artificial point mutations. Finally, the redundancy observed between the QS100 and the entire PDB is the highest, with almost half of the protein complexes in the PDB corresponding to at least one other structure with identical sequence of constituent subunits.

### Adding Symmetry Information to the Classification: An Alternative Hierarchy

**Figure 6:**
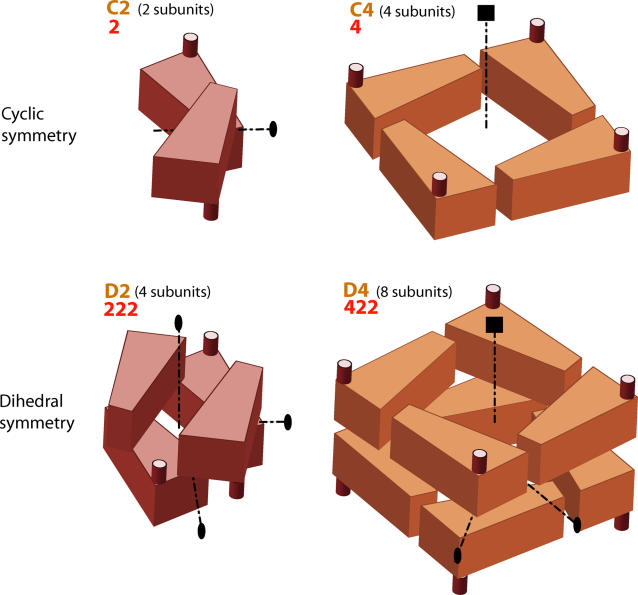
Cyclic and Dihedral Symmetries (C2) Cyclic symmetry: two subunits are related by a single 2-fold axis, shown by a dashed line. An ellipse at the end of the symmetry axis marks a 2-fold axis. Nearly all homodimers have C2 symmetry. C2 symmetry is termed “2” in the crystallographic Hermann-Mauguin nomenclature, shown in red beneath C2. (C4) Cyclic symmetry: four subunits are related by one 4-fold axis. A square at the end of the symmetry axis marks a 4-fold axis. (D2) Dihedral symmetry: four subunits are related by three 2-fold axes. D2 symmetry can be constructed from two C2 dimers. Note the difference between the D2 and C4 symmetries: two symmetry types that both have four subunits. (D4) Dihedral symmetry: eight subunits are related to each other by one 4-fold axis and two 2-fold axes. Note that D4 symmetry can be constructed by stacking two C4 tetramers as shown, or four C2 dimers (not shown).

Knowing the symmetry of a complex confers information about the 3-D arrangement of the subunits that is not provided by the graph representation. For example, there are two symmetric ways to arrange the subunits of a homotetramer. One is with a cyclic symmetry, in which the four subunits are related by a single 4-fold axis, called C4 symmetry, as shown in Figure 6. The other is a dihedral symmetry in which the four subunits are related by three 2-fold axes, called D2 symmetry (Figure 6). A priori, one cannot distinguish the two symmetry types from the graph representation alone. To assess whether the graph representation suffices to account for the spatial arrangement of the subunits, we asked whether QSs might contain complexes with different symmetries.

We calculated the symmetries and pseudosymmetries for all structures, as described briefly above and in detail in Methods. We classify complexes into two categories related to symmetry. We distinguish between complexes that can or cannot be symmetrical on the basis of their polypeptide chain composition. For example, homodimers can be symmetrical, while heterodimers of nonhomologous chains cannot. This is explained in more detail in Methods. Furthermore, QSs with multiple complexes can contain several different symmetry types, while those with just one complex clearly cannot.

We will see below that only a small fraction of QSs contain complexes with different symmetries, which provides support for our use of the 2-D graph representation for comparison of 3-D complexes. In other words, in most cases, a single QS graph represents complexes that all have the same symmetry type.

### The Graph Representation as an Aid to Correctly Identify Biological Units

First, we looked for disagreement in the symmetries amongst complexes within a QS to identify errors or unusual complexes. Among the 841 QSs with a possible symmetry and containing multiple complexes, we found that 109 QSs (13%) contained mixed symmetry types. A manual inspection revealed that 93 of these cases are in fact due to a mix between presence and absence of symmetry in the complexes of each QS. The reason for the absence of symmetry is either biological, for example due to a conformational change (16 cases), or due to an error in the PDB Biological Unit (42 cases). There were also two cases of a false negative result in our symmetry search procedure and 33 ambiguous cases that we were unable to resolve. There are a further 16 QSs with two different symmetry types. Of these, seven are true biological cases, five are errors in the PDB Biological Unit, and three are unresolved. The PDB codes of likely erroneous Biological Units are provided in Table S2A and S2B.

In addition to mixed symmetries, further criteria to filter for errors can be derived from the representation of a protein complex as a graph. For example, in homomeric complexes, in which all the subunits are identical, all chains are expected to have the same number of interfaces. The graph representation allowed us to identify cases where this requirement is not satisfied.

A difference in the number of interfaces of the subunits within a homomeric complex can be biological and is associated with conformational changes in most cases. An example is the hexameric prokaryotic Rho transcription termination factor (PDB 1pv4), which forms an open ring resulting in a linear graph topology in its unbound state [36]. The ring closes upon RNA binding, and so presumably in this state all subunits form two interfaces.

However, in some cases, the asymmetrical graph topology of homomeric complexes corresponds to an error in the definition of the PDB Biological Unit. In Figure 3C, we show four different QS topologies and the number of wrongly defined biological units associated with them. We provide the PDB identifiers of the erroneous cases in Table S2C. In Table S2D we provide the identifiers of 62 possible additional errors found during searches described above, but for which support from literature was not available.

### Comparison of PDB and PQS Biological Units

The PDB and the PQS servers are the only resources that provide information on Biological Units of crystallographic structures. An essential difference between these two resources is that PDB Biological Units are partially manually curated, whereas those from PQS are generated in an entirely automated manner. It is therefore interesting to compare the extent of agreement between the two databases.

Manual inspection and curation of more than 20,000 Biological Units present in both databases is extremely time-consuming. However, we can capture essential differences by comparing the much smaller number of QSTs. We have seen that the PDB Biological Units correspond to 192 QSTs. The PQS yields a slightly higher number of 218 QSTs. When comparing the two sets, we find 155 QSTs common to the PDB and PQS, implying 37 exclusive to the PDB and 68 exclusive to the PQS. We hand-curated the structures exclusive to each database and found that 19 out of the 40 QSTs exclusive to the PDB, and 42 out of 68 QSTs exclusive to the PQS, are likely errors. The accession codes of these structures are shown in Table S2E and S2F. Often, an erroneous Biological Unit in one database is correct in the other. For example, the structure 2dhq, a 3-dehydroquinase composed of 12 identical subunits [37], is found in the correct state in the PQS but has only ten subunits in the PDB Biological Unit. An opposite example is the enzyme MenB from Mycobacterium tuberculosis (1q51), which consists of a homohexamer [38]. Here, the PDB Biological Unit is correct, while in the PQS the enzyme is described as a dodecamer (12 subunits). These examples suggest that a combination of both resources might be a valuable approach for the curation of biological units.

### The Quaternary Structure of Homo-Oligomers beyond the Protein Data Bank

In the section about QSTs, we showed that most complexes in the PDB are small, homomeric, and symmetrical. How general is this result? The PDB is a restricted dataset in which transmembrane proteins, low complexity regions, and disordered regions are underrepresented [39,40], and in which functional biases have also been observed, though structural genomics projects are narrowing the gap [39]. Therefore, we now compare the frequency of homomers in the complete PDB, the PDB QS level, the complete SwissProt, and subsets of human and E. coli proteins in SwissProt.

**Figure 7:**
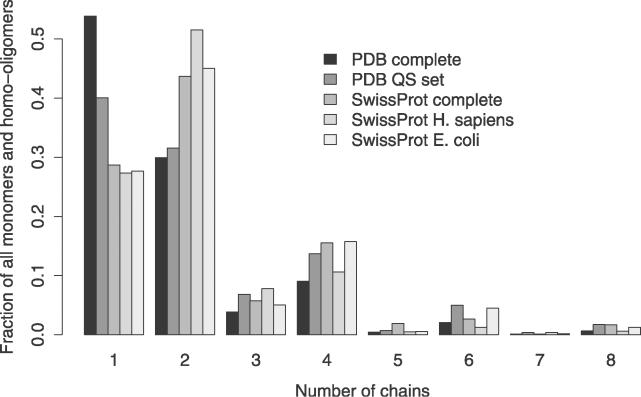
The Size of Homomeric Complexes in the Protein Data Bank and in SwissProt The histogram shows the relative abundances of monomers and homo-oligomers of different sizes in the PDB and in SwissProt. Two PDB sets are shown: the complete set and the nonredundant set of QSs. Three SwissProt sets are shown: the complete SwissProt and the Human and E. coli subsets. The trend in all the sets is similar and highlights the importance of the mechanism of self-assembly, which is linked to many functional possibilities discussed in the text. The oligomeric state of proteins in SwissProt was extracted from the subunit annotation field, and annotations inferred by similarity were not considered.

Interestingly, the trend in the PDB is in close agreement with our observations in SwissProt as shown in Figure 7. In the PDB, we observe 46% of homo-oligomers in the complete set, 60% in the nonredundant set, and between 71% and 73% in SwissProt. Thus, our nonredundant set is more similar to SwissProt than the entire PDB. There is agreement at an even more detailed level in all five datasets: even numbers of subunits are favoured among complexes of size four or more. Homomers with an odd number of subunits can only adopt cyclic symmetries, while even-numbered homomers with four or more subunits can adopt either dihedral or cyclic symmetries [41] (Figure 6). Therefore, the preference for even numbers of subunits suggests that most of these complexes adopt a dihedral symmetry. Indeed, PDB complexes of size four or more with an even number of subunits adopt dihedral symmetries in 80% of the cases and cyclic in 20%. Presumably this is because evolution and stability of dihedral complexes is more favourable than for cyclic complexes. The close agreement between the PDB and SwissProt supports the PDB as a representative set of QSTs.

All five datasets show that homo-oligomerization is very widespread. This could be because it provides simple ways of regulating protein function. It can serve as a sensor of protein concentration or pH at which self-assembly occurs and triggers a function, as in the case of the cell death protease caspase-9 [42]. It can provide cooperativity through an allosteric mechanism, as in the case of the hemoglobin [43]. It can also serve as a template for bringing together proteins and triggering a function, as in the case of the tumor necrosis factor [44]. This is an important message since the recent advances in large-scale mapping of protein complexes by mass spectrometry do not account for the stoichiometry of the subunits. This may project an image of the cell where proteins interact only with other proteins, without taking into account the importance of homo-oligomerization.

It is interesting to note the apparent contradiction between large-scale proteomics data and structural data. In proteomics data, most proteins are interconnected into a “giant component” [45], while in the PDB there are few heteromeric protein complexes, and most are homomeric. This apparent contradiction stems from the fact that small, stable complexes are easiest to crystallize. On the other hand, the goal of proteomics projects is to maximise the coverage and pull out all interactors. The many homomeric complexes of the type observed in the PDB are likely to be at the core of the larger multiprotein complexes seen in proteomics datasets. For example, the 20S proteasome in the PDB consists of homomeric rings [46] and forms the catalytic core of the 26S complex, which contains many more proteins.

### The 3D Complex Database and Web Server

The hierarchical classification of all complexes in the PDB is available as a database on the World Wide Web at http://www.3Dcomplex.org. We pre-computed three classifications, each with and without symmetry information. Note, that even without selecting symmetry as a classification criterion, the symmetry information is still displayed, so that one can see whether a group contains one or several symmetries. Two of the pre-computed classifications start with the QS topology and differ in the combination of sequence identity levels. The third pre-computed classification starts at the QS level, so that all QSs can be viewed on the same page.

It is also possible to select any combination of levels in the classification and browse the result computed on the fly. Combining together different levels in the classification yields different sets of complexes that can be viewed together. For example, when choosing the first and the last level only, QS topologies are linked to all PDB structures. This could be used, for example, to survey all the PDB structures composed of four proteins connected in a particular way.

The database can also be searched by PDB accession code, by SCOP superfamily identifier or domain architecture, by keyword, and by symmetry type. An example of an application of the search facility is a query for all the protein complexes in which one particular domain superfamily participates, in order to learn about the evolution of the interactions of that superfamily. One could also search for a combination of terms, such as transferases that have D2 symmetry.

Besides automatic search and downloading options, manual inspection of complexes is facilitated by the novel visualization mode of representing complexes as graphs. This allows one to analyse and compare many aspects of complexes at a glance that are much more difficult to extract from the standard representations of 3-D structures. For anyone interested in a particular structure, viewing the structure within the 3D Complex classification allows fast comparison with other complexes. For instance, one can quickly gain an overview of the size and pattern of the interfaces in the complex of interest and related complexes.

### Conclusions

Most proteins act in concert with other proteins, forming permanent or transient complexes. Understanding these interactions at an atomic level is only possible through analysis of protein structures. Here we have presented a novel method to describe and compare structures of proteins complexes, which we used to derive a hierarchical classification system.

This hierarchical classification allows us to answer to the question, “How many different complexes exist in the PDB?” Depending on the level of detail, we find from 192 structures at the top level to 12,231 structures at the bottom level of the hierarchy. Which one of these levels is used in an analysis will depend on the type of question addressed.

Considering the top level of the hierarchy, the QSTs, we see a strong bias toward small, homomeric, and symmetrical complexes, and we show that this result can be generalized to SwissProt proteins. We observe that complexes with an even number of subunits are favoured in SwissProt, indicating that dihedral symmetries are more frequent than cyclic symmetries, in the same way as in the PDB. The QS family and QS levels are appropriate nonredundant sets of complexes for many types of analysis. Here we use the QS level for the comparison with SwissProt, and we find that it is in closer agreement than the complete set of complexes.

The remaining levels encompass sequence homology between complexes, ranging from a sequence identity threshold of 20% for QS20 to 100% for QS100, at 10% sequence identity intervals. Using these levels, we explore how four types of similarities between complexes (structural, sequence divergence, point mutation, and identical complexes) relate to each other. At all four levels, we observe the same trend: many complexes are unique, and a few are highly redundant. By integrating these levels with symmetry information, one can address issues such as the sequence threshold at which symmetry type is conserved or broken. By projecting the levels onto each other, the abundance of homologues at different sequence identity thresholds becomes apparent.

We describe the first global framework for analysis of protein complexes of known 3-D structure. The classification will be a starting point for future work aimed at understanding the structure, evolution, and assembly of protein complexes. It is our hope that it will facilitate a better understanding of protein complex space, in the same way SCOP and CATH have played major roles in our understanding of fold space [47]. This is particularly important in the era of structural genomics moving toward solving larger complexes of proteins (e.g.*,* 3D-repertoire, http://www.3drepertoire.org) and with the increasing proteomics data on protein complexes [2].

## Methods

### Comparing protein complexes: The graph alignment procedure.

The graph alignment algorithm developed here is a modified implementation of the A* algorithm [48]. It takes two graphs (*G_a_*, *G_b_*) as input and three tolerance parameters: *M,* the number of label mismatches; *I,* the number of node indels (insertions or deletions); and *E,* the number of edge indels. It returns whether *G_a_* matches *G_b_* allowing for *M*, *I*, and *E*. An additional parameter, *S,* is a score threshold above which a pair of nodes is matched.

The algorithm can be decomposed into four steps: (i) take a node *N_a_i* from *G_a_* at random. (ii) Map it to all the nodes *N_b_j* from *G_b_*. The mappings (*N_a_i*–*N_b_j*) with a valid cost (costs are explained below) are added to the list of mappings denoted as *L*. (iii) Extract from *L* a mapping *m* with the best (lowest) cost and extend it, i.e., take a node of *G_a_* that is not contained in *m* and that is connected to a node in *m;* map it to all the nodes of (*G_b_* , *gap*) that have not been mapped yet, and create a new mapping for each. Add the new mappings with a valid cost (see below) to *L*. (iv) Restart stage 3 *either* until *L* is empty, in which case the two graphs could not be matched, *or* until a mapping *m* contains all the nodes from *G_a_* and *G_b_,* in which case the two graphs are matched. Note that the procedure is exhaustive and therefore does not depend on which node is picked first at random.

Given a mapping *m,* we calculate three costs: (i) *C_M_,* the number of pairs (*N_a_i– N_b_j*) that do not have the same domain architecture, or whose sequence similarity is below the threshold *S*. (ii) C*_I_,* the number of pairs containing a *gap* (*N_a_i*–*gap*). Note that in the current version, gaps cannot be inserted in *G_a_* but only in *G_b_*. (iii) C*_E_,* the number of edge inconsistencies between the mapped nodes.

The mapping is only valid if the costs *C_M_ , C_I_,* and *C_E_* are below or equal to the tolerance parameters *M, I,* and *E,* respectively*.* In the present study, the QST were generated with *M* = number of nodes in *G_a_* and *S* = 0, because homology between nodes is not considered at the QST level. The highest resolution structure of each QST is taken as a representative of the group. Matched complexes are clustered by single linkage to create the groups of complexes that constitute this level of the hierarchy. QS families and QSs were generated with *M* = 0, as the domain architectures have to match perfectly between two complexes, but the sequence identity parameter *S* = 0. The consistency in the attribute “number of genes per domain architecture” was checked prior to the graph comparison. All the other levels (from QS20 to QS100) were generated with *M* = 0 and *S* ranging from 20 to 100. The number of node and edge mismatches tolerated was 0 throughout all levels (*I* = *E* = 0), though this could be loosened in future work.

The graph images on the Web site at http://www.3Dcomplex.org were generated using *GraphViz* [49].

### Finding symmetries in protein complexes.

The process of finding symmetries is performed in three main steps. First, we check whether symmetry can exist in a complex based on its composition in terms of groups of identical or homologous chains. If each group of identical or homologous chains contains an even or odd number of chains (different from one), then symmetry can exist, and the complex is labelled either with the name of the symmetry type found or with NS if no symmetry is found. If we see that no symmetry can exist, e.g., in the case of a heterodimer of nonhomologous subunits, the complex is classified in the *no possible symmetry* (NPS) category, and these complexes are not used in the following steps.

For the next two steps, let's take as an example a complex with two groups of identical chains AB and CD (A is identical to B, C is identical to D, and A is different from C). We first extract the α-carbon of *N* equivalent residues for each group. *N* is limited to 50 but must be larger or equal to 15. Structurally equivalent residues are found using a FASTA sequence alignment [50]. We discarded structures where fewer than 15 common residues were found between homologous chains. At the end of the process, we obtain the coordinates of the α-carbon of at least 15 equivalent residues for each group of identical or homologous chains (Figure S1A).

Next we search for axes and angles of symmetry. First, we centre the coordinates of the structure on its centre of mass (green point in Figure S1B). Then we generate a set of 600 axes shown in Figure S1C as imaginary lines joining the green point and each blue point. We then rotate the structure around each axis by angles ranging from 360/*n* to (180 + (360/*n*)) degrees, by steps of 360/*n,* where *n* is the number of subunits. After each rotation, a distance *d* is measured and is equal to the mean of the Euclidian distances of each atom with its closest structurally equivalent atom. The distance *d* reflects the quality of the superposition associated with each axis and angle. We select the top 2*n* axes and refine each of them to minimize *d*. We retain all axes and angles for which *d* < 7 Å, and we group those separated by less than 25 degrees. Thus, for each structure, we obtain a set of axes and angles of symmetry from which we deduce the symmetry type.

We used the consistency of symmetry assignment as a benchmark for our method: we expect all the structures in the same QS to have the same symmetry. Among the 841 QSs that contain two or more structures with a possible symmetry, we found that 109 contained different symmetry types. This difference could either be true or due to an error in our symmetry search procedure. After manual inspection of these 109 classes, we found that only two errors were due to our procedure. These 109 classes correspond to 2,444 proteins; therefore, we estimate the error rate of the symmetry search procedure to be ∼0.001.

## Supporting Information

Figure S1 Principle of the Symmetry Calculation (A) Within each complex, identical or homologous chains (same N to C terminal domain architecture) are grouped. A set of 50 (and at least 15 for smaller chains) structurally equivalent residues is selected for the chains within each group and represented using the alpha-carbons. The protein shown here is a homohexamer, so there is a single set of equivalent residues.
(B) The coordinates of the set are transformed so that the origin is the centre of mass, shown as a green point.
(C) 600 axes are generated, connecting the green origin to each of the 600 blue points. Rotations of (360/*N*) degrees, where *N* is the number of subunits, are applied around each axis. An RMS deviation is calculated after each rotation. If it is lower than 7 Å, the axis and the angles are retained. The symmetry of the complex is deduced based on the set of retained axes and angles of symmetry.
(63 KB PDF)

Protocol S1 The PDB Biological Unit (29 KB DOC)

Table S1 Effect of Threshold for a Chain–Chain Contact Definition on the Classification The percentage overlap in the table indicates the proportion of structures that stay in the same QS class when varying the threshold for a chain–chain contact definition. The threshold value corresponds to the number of residues contributed by both chains. The high overlap shows that our methodology is robust.
(28 KB DOC)

Table S2 List of Likely Errors in the Biological Unit Reconstruction PDB accession codes in parentheses are redundant complexes for which the same error was found. For each PDB structure, the number of chains in the current Biological Unit is given, as well as our own suggestion for correction of the prediction. In cases where the current and suggested number of chains is the same, we believe the Biological Unit should contain different interfaces with the same number of chains.
(A) Errors were found upon manual inspection of groups of protein complexes in the same QS that contained some complexes for which no symmetry was detected and other complexes with symmetry. The number of likely errors in this table is 68.
(B) Errors were found upon manual inspection of protein complexes in the same QS that had different symmetry types. The number of likely errors in this table is 13.
(C) Errors were found by looking manually at peculiar QS Topologies, where identical subunits did not have the same number of contacts. The groups correspond to the four different topologies shown in Figure 4C. The number of likely errors in this table is 51.
(D) List of possible errors found during searches described for the three tables above, but for which support from sources such as literature was not available. The number of possible errors is 62.
(E) Errors were found during the comparison process between topologies found in PDB and PQS. The following list comes after curation of the topologies that are exclusive to PDB.
(F) Errors were found during the comparison process between topologies found in PDB and PQS. The following list comes after curation of the topologies that are exclusive to PQS.
(63 KB DOC)

## Abbreviations

PDB: Protein Data Bank
PQS: Protein Quaternary Structure
QS: Quaternary Structure
QSF: Quaternary Structure Family
QST: Quaternary Structure Topology
