# Supplementary material for: 3D Complex: A Structural Classification of Protein Complexes
Source: PLoS Comput Biol. 2006 Nov 17;2(11):e155. doi: 10.1371/journal.pcbi.0020155 (PMC1636673; doi:10.1371/journal.pcbi.0020155)
Supplement: Supplementary file 1 [file pcbi.0020155.sg001.pdf]

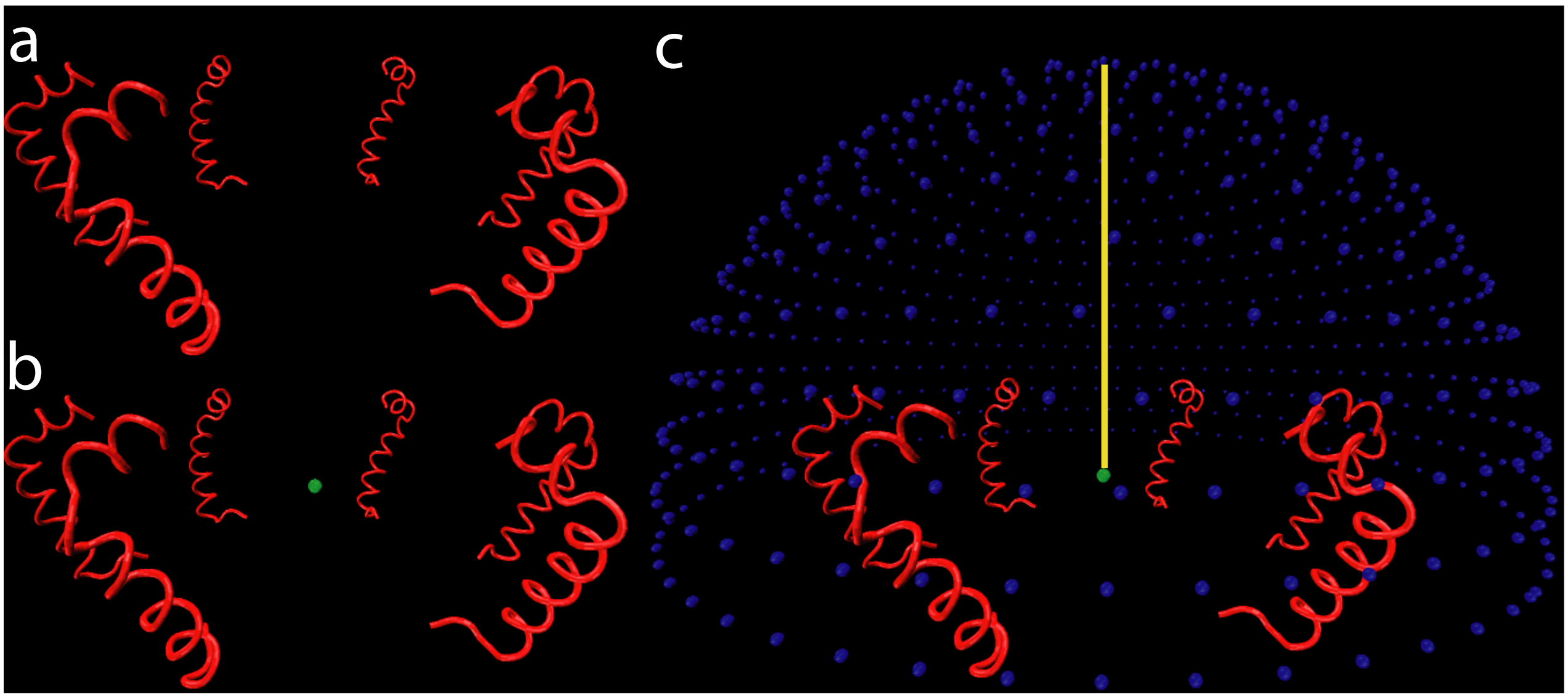
a
b
c
