# Supplementary material for: 3D Complex: A Structural Classification of Protein Complexes
Source: PLoS Comput Biol. 2006 Nov 17;2(11):e155. doi: 10.1371/journal.pcbi.0020155 (PMC1636673; doi:10.1371/journal.pcbi.0020155)
Supplement: Supplementary file 2 [file pcbi.0020155.sd001.doc]

## The PDB Biological Unit

X-ray crystallography is the most common method used to gain information about protein structure. Structures obtained with this method correspond to an asymmetric-unit (ASU) *i.e.* the minimal unit from which the whole crystal can be reconstructed using symmetry operations. Importantly, the protein(s) contained in the ASU may not be in their native functional oligomeric state. For example, an ASU may contain a single copy of a protein that actually forms a homohexamer *in vivo*, or it might contain a dimer of a protein that exists as a monomer *in vivo*. This is why, in addition to the coordinates of the ASU, the Protein Data Bank makes available the coordinates of the Biological Unit, which is believed to contain the structure of the protein in its native state. For structures released up to 1998, the Biological Units were created automatically using the Protein Quaternary Structure server (PQS, (Henrick and Thornton 1998)). For structures from 1998 onwards, which are the majority of structures in PDB, the Biological Unit is manually curated and inferred based on the expertise of the authors of the structure as well as the PDB curators.
