# Supplementary material for: 3D Complex: A Structural Classification of Protein Complexes
Source: PLoS Comput Biol. 2006 Nov 17;2(11):e155. doi: 10.1371/journal.pcbi.0020155 (PMC1636673; doi:10.1371/journal.pcbi.0020155)
Supplement: Supplementary file 3 [file pcbi.0020155.st001.doc]

| **Threshold value** | **8** | **10** | **12** |
| --- | --- | --- | --- |
| **Overlap of the QS classes with those obtained at 10 interface residues** | 95.4% | 100% | 96.1% |
