# Supplementary material for: 3D Complex: A Structural Classification of Protein Complexes
Source: PLoS Comput Biol. 2006 Nov 17;2(11):e155. doi: 10.1371/journal.pcbi.0020155 (PMC1636673; doi:10.1371/journal.pcbi.0020155)
Supplement: Supplementary file 4 [file pcbi.0020155.st002.doc]

**Table S2.** List of likely errors in the Biological Unit reconstruction.

PDB accession codes in parentheses are redundant complexes for which the same error was found. For each PDB structure, the number of chains in the current Biological Unit is given, as well as our own suggestion for correction of the prediction. In cases where the current and suggested number of chains is the same, we believe the Biological Unit should contain different interfaces with the same number chains.

**a.** Errors were found upon manual inspection of groups of protein complexes in the same Quaternary Structure which contained some complexes for which no symmetry was detected and others with a symmetry. The number of likely errors in this table is 68.

| PDB ID | No. chains in  current Biological Unit | No. chains  suggested for Biological Unit |
| --- | --- | --- |
| 1jnp | 2 | 2 |
| 1h4x | 2 | 1 |
| 1olo | 2 | 6 |
| 1b0u | 2 | 2 |
| 1jqn | 4 | 4 |
| (1,2,5,6,7,8,9)jdx | 2 | 2 |
| 1smo (1pew) | 2 | 1 |
| 1mze (1mzf) | 2 | 2 |
| 1md6 | 2 | 1 |
| 1r3(q,r,s,t,v,w,y) | 2 | 2 |
| 1b9c | 4 | 2 |
| 1lm5 (1lm7) | 2 | 1 |
| 1d7x (1b3d) | 2 | 1 |
| 1eo6 | 2 | 1 |
| 1l9n (1l9m) | 2 | 1 |
| 1h5y | 2 | 1 |
| 1dv7 | 2 | 2 |
| 1b6r | 2 | 2 |
| 1qkr | 2 | 1 |
| 1gze | 4 | 1 |
| 1fr8 | 2 | 1 |
| 1c4k | 2 | 2 |
| 1oj6 | 4 | 4 |
| 1fxs (1bws) | 2 | 2 |
| 1dd7 | 2 | 1 |
| 1fx2 (1fx4) | 2 | 1 |
| 1ggg | 2 | 1 |
| 1gmp (1rgf,1rgg) | 2 | 1 |
| 1iwo | 2 | 1 |
| 1aoc | 2 | 1 |
| 1hzb | 2 | 1 |
| 1gu8 | 2 | 2 |
| 1ot6 (1ot9) | 2 | 2 |
| 1ecx | 2 | 2 |
| 1bk4 | 2 | 4 |
| 1qnh | 2 | 1 |
| 1kmz | 2 | 2 |
| 1jia | 2 | 2 |
| 1ml1 (1dkw) | 2 | 1 |
| 1jyl | 2 | 1 |
| 1mz0 | 2 | ? |
| 1h07 | 2 | 1 |
| 1oqz | 2 | 2 |
| 1sdy | 4 | 2 |
| 1srd | 2 | 2 |

**b**. Errors were found upon manual inspection of protein complexes in the same QS that had different symmetry types. The number of likely errors in this table is 13.

| PDB ID | Current symmetry type (No. chains) | Suggested symmetry type (No. chains) |
| --- | --- | --- |
| 1cvi | C2(4) | C2(2) or D2(4) |
| 1key | C4(4) | D4(8) |
| 1j1j | C2(4) | D4(8) |
| 1ufh | C2(4) | D2(4) |
| 1vwi | C2(4) | D2(4) |
| 1vwl | C2(4) | D2(4) |
| 1moy | C2(4) | D2(4) |
| 1mm9 | C2(4) | D2(4) |
| 1qma | D2(4) | C2(2) |
| 1gy7 | C2(4) | C2(2) |
| 1gyb | C2(4) | C2(2) |
| 1ocv | C2(4) | C2(2) |
| 1s5a | C2(4) | C2(2) |

**c**. Errors were found by looking manually at peculiar QS Topologies, where identical subunits did not have the same number of contacts. The groups correspond to the four different topologies shown in Figure 4C. The number of likely errors in this table is 51.

| **PDB ID** | No. Chains in  Current Biological Unit | No. Chains  Suggested for Biological Unit |
| --- | --- | --- |
| Group 1 |  |  |
| 1b5e (1b5d) | 4 | 2 |
| 1n1q | 4 | 2/12 |
| 1l5s | 4 | 2 |
| 1eqr | 4 | 2 |
| 1oa8 | 4 | 2 or 8 |
| 1hf2 | 4 | 2 |
| 1h9u | 4 | 8 |
| 1gzf | 4 | ? |
| Group 2 |  |  |
| 1bgt | 3 | 2 |
| 1g1q | 3 | 1 or 2 |
| 1od4 | 3 | 2 |
| 1tsr (1tup) | 3 | 2 or 4 |
| 1qvr | 3 | 6 |
| 1oms | 3 | 2 |
| 2lzk | 3 | 2 or 4 |
| 1am7 | 3 | 1 |
| 1gmy | 3 | 1 |
| 1juh | 3 | 2 |
| 1h6r | 3 | 1 or 2 |
| 1h65 (1vkj) | 3 | 1 or 2 |
| 1gp7 | 3 | 2 |
| 1rgh | 3 | 2 |
| 1gwc | 3 | 2 |
| 1tui | 3 | 3 |
| 1rv1 | 3 | 2 |
| 1co9 | 3 | 6 |
| Group 3 |  |  |
| 1gqg (1gqh,1h1i,1h1m) | 4 | 2 |
| 1gy8 (1dir, 1gz6) | 4 | 2 |
| 1gte (1gt8,1gth) | 4 | 2 |
| 1hl4 (1srd, 1sdy) | 4 | 2 |
| 2oat (1oat) | 4 | 2 |
| 1mou | 4 | 2 |
| 1ev9 (1ev4) | 4 | 2 |
| 1oa8 | 4 | 2 or 8 |
| Group 4 |  |  |
| 1oa2 | 6 | 1 or 2 |
| 1ovv | 6 | 2 or 4 |
| 1odb | 6 | 2 |

**d.** List of possible errors found during searches described for the three tables above, but for which support from sources such as literature was not available. The number of possible errors is 62.

1s7o

1psq

1b47

1hhs

1rq0

1jnp

1q8b

1k68

1u9d

1fp3, 1trm, 1svp, 2cga, 1dle

1l4i

1o81,1oc8

1bxt

1ff4

1oqz

1gy8,1dir

1bdy

1hmk,137l,1ckg,1b52,1b2k

1bp6,1bpj

1ql3,1gu2

1obo

1c76,1c77

1fbq,1fbu,1fbs

1sb7

1pnv

1rtv

1qpl,1fki

1j3b

6pah

1db2

1odz,1aq0

1bim,1hrn,1bil

1tiy

1e4y

1ilr

1x11,1aqc,1uef,1p5t

1agq

2hlc

1hc7

2rsl

**e.** Errors were found during the comparison process between topologies found in PDB and PQS. The following list comes after curation of the topologies that are exclusive to PDB.

1jzn

1o9k

1atj

1i3r

1gyt

1ha7

1gt7

2dhq

1e9s

1b0c

1vf7

1nlx

1gqm

1f4s

1ei7

1t9g

1gqo

1t5e

1ods

1l0l

1h6d

1h2i

**f.** Errors were found during the comparison process between topologies found in PDB and PQS. The following list comes after curation of the topologies that are exclusive to PQS.

1df8

1ltr

1jth

1bdf

1nkz

1uad

1ft8

1fay

1jn1

1vwj

1azz

1pgr

1f9n

1sfc

1cax

1pvo

1jkj

1eg9

1gug

1v4l

1ml0

1dd4

1mva

1b4a

1fx3

1gto

1dd3

1pv4

1i7z

1j8d

1mab

1nxk

3fru

1xxb

1fnw

1n4p

1f2d

1m7w

1b47

1fak

1q51

1cw3
